# Gut microbiota during pregnancy: a bibliometric analysis of global research trends and collaborative networks

**DOI:** 10.3389/fmicb.2025.1708359

**Published:** 2025-12-10

**Authors:** Ailing Chen, Zouqing Luo, Jinqiu Zhang

**Affiliations:** 1 Research Institute for Reproductive Health and Genetic Diseases, Women’s Hospital of Jiangnan University, Wuxi Maternity and Child Health Care Hospital, Wuxi, China; 2 Department of Obstetrics, Women’s Hospital of Jiangnan University, Wuxi Maternity and Child Health Care Hospital, Wuxi, China; 3 Department of Pathology, Women’s Hospital of Jiangnan University, Wuxi Maternity and Child Health Care Hospital, Wuxi, China

**Keywords:** gut microbiota during pregnancy, Web of Science Core Collection, Scopus, bibliometric analysis, gestational diabetes mellitus, maternal-infant health, gut-brain axis

## Abstract

**Background:**

Investigating gut microbiota during pregnancy is vital for understanding maternal–infant health, pregnancy-related disease mechanisms, and offspring development. While research in this field has grown rapidly, systematic analyses of global trends, collaborative networks, and thematic evolution remain limited. This bibliometric study maps the developmental landscape of “gut microbiota during pregnancy,” identifying research priorities and future directions.

**Methods:**

A bibliometric analysis of pregnancy and gut microbiota studies (1991–2025) was conducted using the Web of Science Core Collection and Scopus databases. Publications were analyzed using bibliometrix, VOSviewer, and CiteSpace to evaluate publication trends, research contributions, collaboration networks, keyword co-occurrence patterns, and thematic evolution.

**Results:**

The analysis encompassed 5,432 (Web of Science Core Collection) and 5,542 (Scopus) publications, with an annual growth rate exceeding 15%. Research output has grown exponentially since 2014. The China and United States were the most productive countries, with the United States demonstrating the highest total citations and a central role in global collaborative networks. Key influential institutions included the University of Turku, University College Cork, and the Chinese Academy of Sciences. Leading authors were Collado, Maria Carmen; Tain, You-Lin; and Cryan, John F. The research was highly interdisciplinary, spanning microbiology, nutrition, immunology, and medicine. Core journals disseminating knowledge were *Nutrients, Frontiers in Microbiology,* and *Gut Microbes*. High-impact and co-cited references established the knowledge foundation, focusing on maternal microbiome remodeling, delivery mode’s impact, and the gut-brain axis. Keyword analysis revealed a thematic evolution from initial descriptive studies of microbial composition to recent investigations into mechanisms linking microbiota to gestational diabetes mellitus, preeclampsia, preterm birth, and neurodevelopmental outcomes via the gut-brain axis.

**Conclusion:**

This study presents an integrative bibliometric analysis of global research on gut microbiota during pregnancy, delineating its rapid evolution and current intellectual structure. The field has matured from descriptive ecology to mechanistic and translational research, with strong international collaboration and interdisciplinary integration. The identified research fronts, including the interplay between microbial dysbiosis and specific pregnancy complications, as well as the influence of the maternal gut microbiome on offspring neurodevelopment, represent promising avenues for future investigation.

## 1 Introduction

Pregnancy is a distinct phase in a woman’s life characterized by dynamic physiological and metabolic adaptations (Gangakhedkar and Kulkarni, 2021). During this phase, the maternal body undergoes extensive immune, hormonal, and metabolic transformations to maintain pregnancy and ensure the fetus develops optimally (Miko et al., 2022). In recent years, advances in microbiome research technologies, particularly the widespread adoption of high-throughput sequencing and integrated multi-omics analytical approaches (Liu et al., 2024; Tang et al., 2024), have positioned the gut microbiota as a focal point of interdisciplinary research bridging life sciences and clinical medicine. Its critical role in pregnancy-related health and disease has garnered increasing attention.

Substantial evidence demonstrates that the structural composition, diversity, and functional dynamics of the maternal gut microbiota evolve significantly throughout gestation (Di Simone et al., 2020; Liu X. et al., 2022; Liu Z. et al., 2022). These shifts are intricately linked to key physiological processes, including immune modulation at the maternal-fetal interface (Adamczak et al., 2024; Faas and Smink, 2025), nutrient metabolism and energy homeostasis (Fu et al., 2024). Notably, gut microbiota dysbiosis has been implicated in the pathogenesis of various pregnancy complications, such as gestational diabetes mellitus (GDM) (Gupta et al., 2024; Li H. et al., 2024; Li Y. et al., 2024; Li J. et al., 2024; Li S. et al., 2024; Sohrabi et al., 2025; Wang et al., 2018), preeclampsia (Jin et al., 2022; Tang et al., 2022; Wang W. et al., 2020; Wang J. et al., 2020), and preterm birth (Kim and Youn, 2024; Uchida et al., 2025). Furthermore, maternal gut microbiota influences offspring health through vertical transmission and microbial metabolites, profoundly shaping the maturation of the infant’s immune system (Cheng et al., 2021; Thompson et al., 2022) metabolic programming (Thompson et al., 2022; Xue et al., 2022), and neurocognitive developmental trajectories (Di Gesu et al., 2022; Liu et al., 2025; Zhao et al., 2021). These findings underscore its pivotal role in determining long-term offspring health outcomes.

As research on gut microbiota during pregnancy intensifies, global scientific output has surged exponentially. Investigations have expanded from foundational descriptions of microbial composition to mechanistic explorations, clinical translation, and the development of targeted intervention strategies, reflecting broad recognition of this field’s academic and clinical significance. However, the sheer volume of literature presents challenges for researchers. Conventional review methodologies, constrained by subjectivity, limited scope, and inadequate capacity to process large-scale data, struggle to comprehensively map the field’s intrinsic developmental patterns. Bibliometrics, an interdisciplinary framework integrating mathematics, statistics, and computational science, enables quantitative and visual analysis of scholarly data to delineate domain-specific productivity, collaboration networks, knowledge foundations, and thematic evolution (Lazarides et al., 2025). By identifying high-impact contributors, mapping global collaborations, and detecting emerging trends, bibliometric analysis provides an evidence-driven foundation for optimizing research priorities and fostering innovation.

Although prior studies have examined bibliometric features of gut microbiota in specific diseases or life stages, no systematic investigation has yet elucidated the knowledge architecture and developmental trajectory of “gut microbiota during pregnancy” as a cross-disciplinary domain. This study addresses this gap by employing multidimensional bibliometric techniques. By applying bibliometric techniques and leveraging the bibliometrix software package, CiteSpace, and VOSviewer visualization tools, it conducts a systematic analysis of the literature related to the intestinal microbiota during pregnancy, which is indexed in the Web of Science Core Collection (WoSCC) and Scopus databases from 1991 to 2025. WoSCC and Scopus are globally recognized as high-quality academic databases, with all included literature undergoing rigorous peer review and screening processes. Empirical research by Zhu and Liu (2020) demonstrates that both databases exhibit high usage frequencies in academic publications, reflecting their practical utility as research tools. Furthermore, a longitudinal comparison reveals that while each database demonstrates distinct advantages in disciplinary coverage, both adequately fulfill the requirements for bibliometric analysis (Harzing and Alakangas, 2016). Our analysis encompasses: (1) temporal trends in annual publication output; (2) distributions of productivity and influence across countries/regions, institutions, and authors; (3) topology of collaborative networks; (4) interdisciplinary convergence patterns; and (5) thematic evolution revealed through keyword co-occurrence networks, burst term detection, and temporal mapping of research frontiers. By elucidating the developmental landscape of this field, our findings offer actionable insights for researchers to navigate evolving hotspots, identify emerging opportunities, and guide clinical practice, thereby contributing to the optimization of scientific and translational priorities.

## 2 Methods

### 2.1 Data collection and retrieval strategy

**Figure 1:**
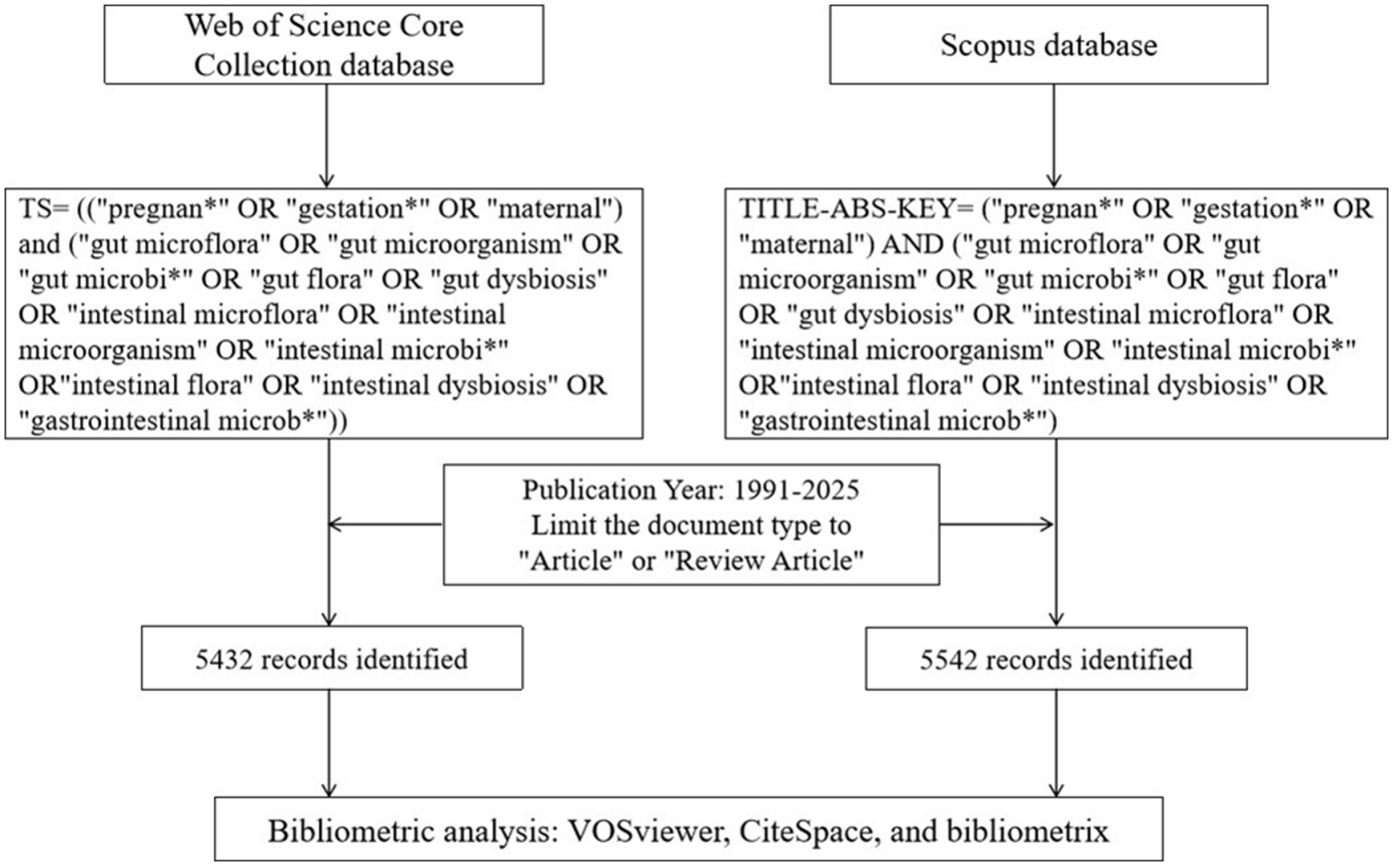
Data retrieval and analysis process.

To ensure comprehensive coverage and data accessibility, a systematic literature search was conducted on September 10, 2025, in the WoSCC and Scopus databases. The specific search strategy is illustrated in Figure 1. The search query for WoSCC was constructed using the “Topic” (TS) field, which includes title, abstract, author keywords, and Keywords Plus: TS = ((“pregnan*” OR “gestation*” OR “maternal”) and (“gut microflora” OR “gut microorganism” OR “gut microbi*” OR “gut flora” OR “gut dysbiosis” OR “intestinal microflora” OR “intestinal microorganism” OR “intestinal microbi*” OR”intestinal flora” OR “intestinal dysbiosis” OR “gastrointestinal microb*”)).

For Scopus, the search was built using the “TITLE-ABS-KEY” (title, abstract, keywords) field: TITLE-ABS-KEY = (“pregnan*” OR “gestation*” OR “maternal”) AND (“gut microflora” OR “gut microorganism” OR “gut microbi*” OR “gut flora” OR “gut dysbiosis” OR “intestinal microflora” OR “intestinal microorganism” OR “intestinal microbi*” OR”intestinal flora” OR “intestinal dysbiosis” OR “gastrointestinal microb*”).

The literature types were limited to “Articles” and “Review Articles” published between 1991 and 2025, with no language restrictions. During the data screening process, 5,432 and 5,542 topic-relevant records were identified from WoSCC and Scopus, respectively. The following data were extracted and exported separately from each database for independent analysis: publication year, authors, subject categories, affiliations, journal names, countries/regions, keywords, and references. Additionally, 3,837 overlapping publications were identified between the two databases.

### 2.2 Data analysis

Bibliometric analysis was performed using the bibliometrix package (version 5.1.1) within the R programming environment (version 4.5.1), supplemented by VOSviewer (v1.6.19) and CiteSpace (v6.2. R3). Initial analyses utilized the built-in database statistics and the bibliometrix package to examine publication distributions, countries/regions collaborations, authors, and the top 10 highly cited and co-cited references. VOSviewer was further employed to extract and visualize institutional and keyword clustering networks. CiteSpace was used to perform country co-occurrence analysis, reference co-citation clustering analysis, and to generate a keyword timeline map. Additionally, a burst detection analysis was conducted for the top 30 keywords with the strongest citation bursts.

## 3 Results

### 3.1 Annual global publication outputs on “gut microbiota during pregnancy”

**Figure 2:**
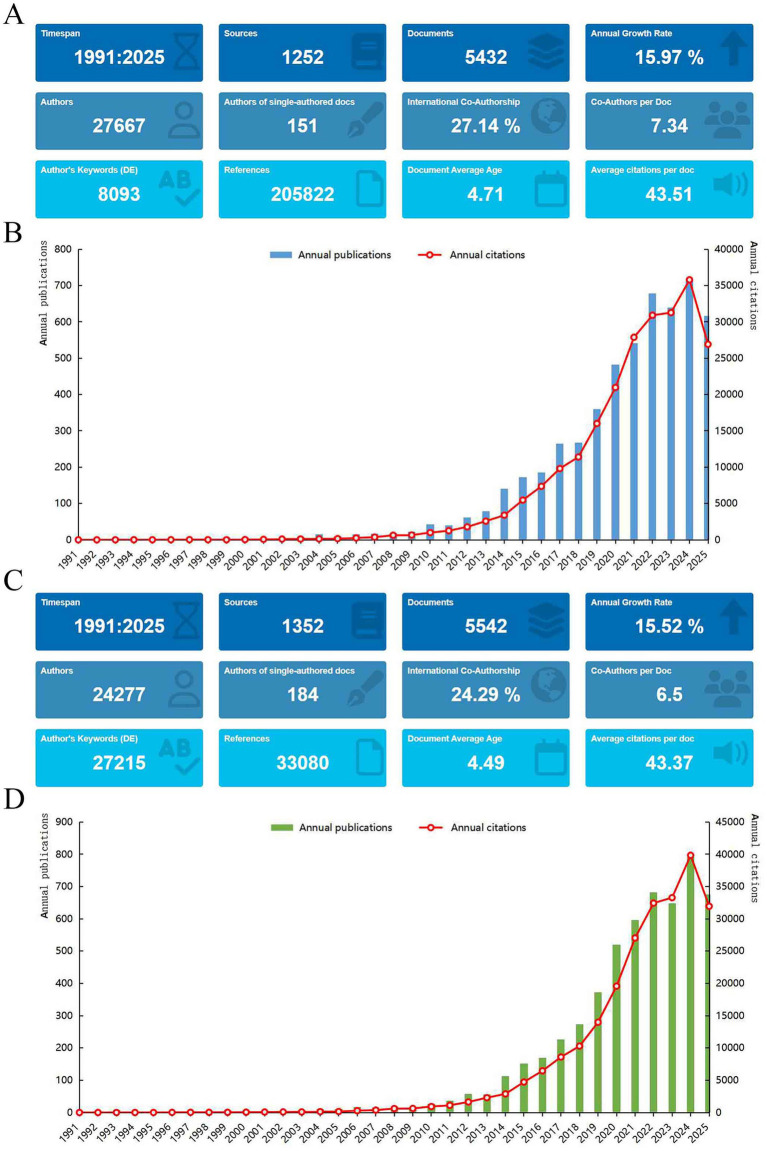
Publication metrics and temporal trends. **(A)** Bibliometric overview from WoSCC database; **(B)** annual publication distribution in WoSCC database; **(C)** bibliometric overview from Scopus database; **(D)** annual publication distribution in Scopus database.

Analysis of the WoSCC and Scopus databases identified 5,432 and 5,542 publications, respectively (Figures 2A,C). There were 3,837 overlapping publications between the two databases. Figures 2B,D illustrate the annual publication trends, which show a high degree of consistency between the two databases, with annual growth rates of 15.97 and 15.52%, respectively. During the early stage (1991–2006), the annual publication count was generally below 20, and annual citations were fewer than 300. A phase of steady development occurred between 2007 and 2013, with a gradual increase in annual publications. Exponential growth has been observed since 2014, with both databases recording over 700 publications in 2024, reflecting the sustained high impact and growing academic interest in maternal and infant health research.

### 3.2 Distribution and co-authorship of countries/regions

**Figure 3:**
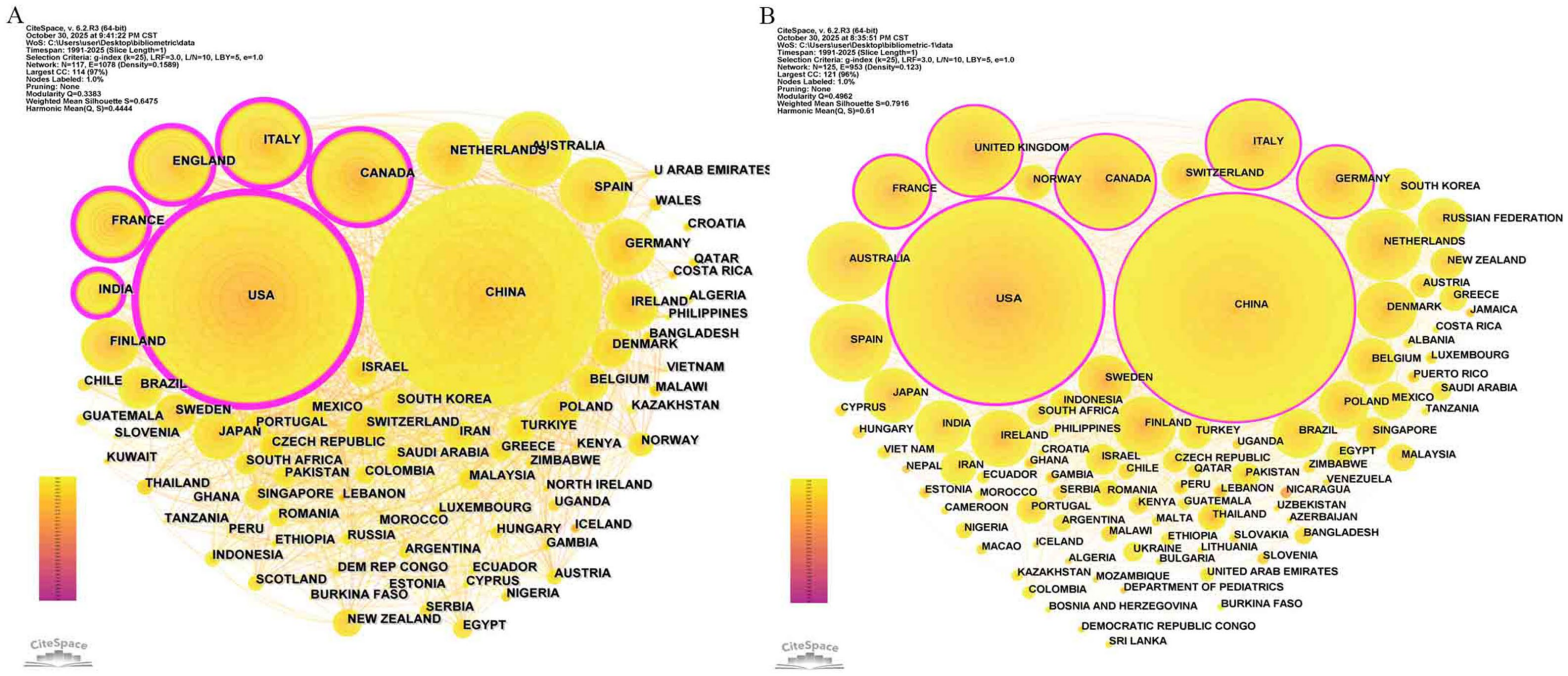
International collaboration patterns. **(A)** Countries/regions network from WoSCC database; **(B)** Countries/regions network from Scopus database.

From 1991 to 2025, research on “gut microbiota during pregnancy” has garnered widespread global attention. In the WoSCC database (Table 1), China took the lead, with 1,561 publications (accounting for 21.67%), a total of 33,826 citations (average of 21.67 citations per article), an H-index of 80, a total link strength of 443 in the VOSviewer collaboration strength analysis, and a betweenness centrality of 0.06 in the CiteSpace collaboration network analysis (Figure 3A). The United States ranked second, with 1,511 publications (accounting for 27.82%), a total of 96,109 citations (average of 63.61 per article), an H-index of 144, a total link strength of 1,018, and a betweenness centrality of 0.3 in the CiteSpace collaboration network analysis, highlighting its advantages in the quality of basic research. Canada ranked third with 342 publications (6.3%), a total of 22,126 citations (average of 64.7 per article), an H-index of 75, a total link strength of 352, and a betweenness centrality of 0.14, indicating its role as a bridge in high-quality research and regional collaboration. England (total link strength 511, betweenness centrality 0.18) and France (betweenness centrality 0.2) emerged as key nodes in global scientific collaboration due to their high collaboration strength.

**Table 1:** Top 10 most productive countries/regions in “gut microbiota during pregnancy” research.

Rank	Country	Publications *n* (%)	Total citations	Average citations	H-Index	Total link strength	Centrality
Web of Science Core Collection database							
1	China	1,561 (28.7)	33,826	21.67	80	443	0.06
2	USA	1,511 (27.82)	96,109	63.61	144	1,018	0.3
3	Canada	342 (6.3)	22,126	64.7	75	352	0.14
4	Australia	295 (5.43)	14,482	49.09	61	313	0.06
5	England	276 (5.08)	16,823	60.95	61	511	0.18
6	Italy	268 (4.93)	14,476	54.01	57	302	0.1
7	Spain	246 (4.53)	14,655	59.57	57	314	0.06
8	Netherlands	242 (4.46)	14,703	60.76	58	397	0.08
9	France	236 (4.34)	9,739	41.27	53	233	0.2
10	Germany	197 (3.63)	11,437	58.06	52	311	0.08
Scopus database							
1	China	1729 (31.20)	36,944	21.37	90	428	0.12
2	USA	1,436 (25.91)	101,868	70.94	149	996	0.5
3	Canada	324 (5.85)	19,282	59.51	70	329	0.13
4	United Kingdom	306 (5.52)	20,643	67.46	72	469	0.17
5	Italy	300 (5.41)	19,825	66.08	64	292	0.13
6	Australia	265 (4.78)	13,932	52.57	59	252	0.04
7	Spain	240 (4.33)	15,628	65.12	59	256	0.08
8	France	234 (4.22)	12,730	54.4	56	250	0.21
9	Netherlands	230 (4.15)	18,773	81.62	66	332	0.04
10	Germany	198 (3.57)	14,602	73.75	56	327	0.16

In the Scopus database (Table 1), China surpassed the United States with 1,729 publications (31.20%) compared to 1,436 (25.91%), a total of 36,944 citations (average of 21.37 per article), an H-index of 90, a total link strength of 428, and a betweenness centrality of 0.12 (Figure 3B), reflecting its leading output volume and high activity in open-access journals. The United States maintained a total of 101,868 citations (average of 70.94 per article), an H-index of 149, a total link strength of 996, and a betweenness centrality of 0.5, underscoring its leading position in high-quality research and collaborative networks. The United Kingdom (total link strength 469, betweenness centrality 0.17) and Germany (betweenness centrality 0.16) served as core nodal countries in Scopus due to their strong interdisciplinary collaboration.

Both WoSCC and Scopus identified the United States, China, and Canada as the top three most productive countries. The United States consistently ranked in the top two across both databases, demonstrating stable research quality and collaboration. China led in output volume in Scopus but still requires improvement in average citations and H-index. Canada ranked in the top three in both databases, reflecting its sustained research investment.

### 3.3 Distribution of research institutions and authors

#### 3.3.1 Institutional distribution

The distribution of institutions and the cooperation network were analyzed using VOSviewer. As shown in Table 2, in the WoSCC database, the University of Turku ranked first with 100 publications (1.84%), a total of 10,515 citations (average of 105.15 per article), and a total link strength of 115. The Chinese Academy of Sciences (86 publications, 2,316 total citations) and University College Cork (86 publications, 6,623 total citations) tied for second place. The University of Colorado ranked fourth with 82 publications and an average of 114.02 citations per article, reflecting its high per-article quality. The University of Toronto (78 publications, total link strength 161) stood out for its extensive international collaboration network.

**Table 2:** Top 10 most productive institutions in “gut microbiota during pregnancy” research.

Rank	Institution	Countries/regions	Publications *n* (%)	Total citations	Average citations	Total link strength
Web of Science Core Collection database						
1	University of Turku	Finland	100 (1.84)	10,515	105.15	115
2	Chinese Academy of Sciences	China	86 (1.58)	2,316	26.93	86
3	University College Cork	Ireland	86 (1.58)	6,623	77.01	80
4	University of Colorado	USA	82 (1.51)	9,350	114.02	62
5	University of Copenhagen	Denmark	80 (1.47)	3,301	41.26	74
6	University of Toronto	Canada	78 (1.44)	5,411	69.37	161
7	China Agricultural University	China	71 (1.31)	1,475	20.77	29
8	Chang Gung University	China (Taiwan)	68 (1.25)	1,618	23.79	147
9	Harvard Medical School	USA	67 (1.23)	2,979	44.46	107
10	University of Melbourne	Australia	67 (1.23)	2,925	43.66	133
Scopus database						
1	Apc Microbiome Ireland	Ireland	105 (1.89)	10,593	100.89	173
2	Københavns Universitet	Denmark	80 (1.44)	5,020	62.75	53
3	University College Cork	Ireland	79 (1.43)	7,994	101.19	147
4	Turun Yliopisto	Finland	69 (1.25)	9,476	137.33	71
5	Chang Gung University College of Medicine	China (Taiwan)	66 (1.19)	1,640	24.85	143
6	Csic – Instituto De Agroquímica Y Tecnología De Alimentos (Iata)	Spain	66 (1.19)	5,530	83.79	45
7	Wageningen University & Research	Netherlands	62 (1.12)	6,481	104.53	58
8	Chang Gung Memorial Hospital	China (Taiwan)	61 (1.1)	1,328	21.77	139
9	Moorepark Food Research Centre	Ireland	61 (1.1)	4,898	80.30	119
10	University of Alberta	Canada	61 (1.1)	5,698	93.41	137

In the Scopus database, APC Microbiome Ireland led with 105 publications (1.89%), a total of 10,593 citations (average of 100.89 per article), and a total link strength of 173. University College Cork (79 publications, average of 101.19 citations per article) ranked third. Turun Yliopisto (69 publications) ranked fourth, with a notably high average of 137.33 citations per article. A cross-database comparison revealed that University College Cork ranked among the top three in both databases. Overall, the field has formed a collaborative research landscape centered on institutions in North America, Europe, and East Asia, with broad participation from multiple regions.

**Figure 4:**
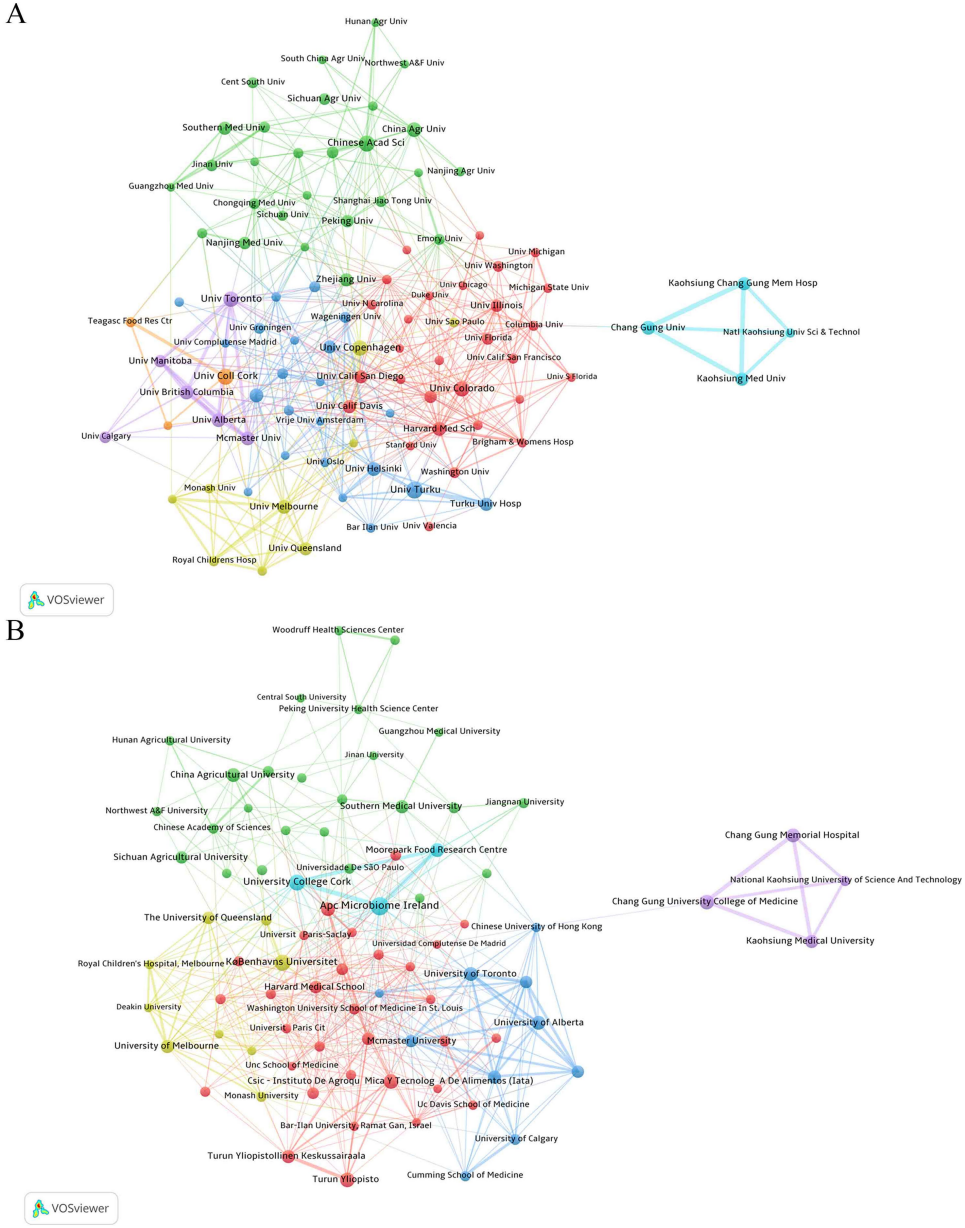
Institutional collaboration networks. **(A)** WoSCC database; **(B)** Scopus database.

As shown in Figure 4A, the top publishing institutions in the WoSCC database were grouped into seven distinct clusters. The red cluster primarily consisted of institutions from the United States, Denmark, and Sweden, including the University of Colorado, the University of Copenhagen, and Harvard Medical School. The green cluster was mainly composed of institutions from China and Ireland, such as the Chinese Academy of Sciences, University College Cork, China Agricultural University, and Zhejiang University. The yellow cluster included Australian institutions like the University of Melbourne and the University of Western Australia. The purple cluster comprised Canadian institutions, including the University of Toronto and the University of Alberta. The blue cluster consisted mainly of institutions from Finland, the Netherlands, and Spain, such as the University of Turku, the University of Helsinki, and Turku University Hospital. The light blue cluster was represented by Chang Gung University, and the orange cluster by Bar-Ilan University.

Based on the Scopus database (Figure 4B), the institutional collaboration network was divided into six clusters. The red cluster exhibited multinational collaboration, including institutions from the United States (e.g., Harvard Medical School; Karolinska Institutet), Sweden, Finland, Spain, and Israel (Bar-Ilan University). The green cluster was concentrated in Chinese institutions, including China Agricultural University, the Chinese Academy of Sciences, and Sichuan Agricultural University. The blue cluster was dominated by Canadian institutions, such as the University of Toronto, McMaster University, and the University of Alberta. The yellow cluster integrated Australian (University of Melbourne, Monash University) and Danish (Københavns Universitet; Det Sundhedsvidenskabelige Fakultet) institutions. The light blue cluster was centered in Ireland, including APC Microbiome Ireland, University College Cork, and Moorepark Food Research Centre. The purple cluster was represented by Chang Gung University College of Medicine.

#### 3.3.2 Author distribution

Analysis of author distribution using the bibliometrix package in R revealed that in the WoSCC database, the most prolific author was Collado, Maria Carmen from the Instituto de Agroquímica y Tecnología de Alimentos, with 75 publications (1.38% of total publications), followed by Tain, You-Lin from Kaohsiung Chang Gung Memorial Hospital with 61 publications (1.12%), and Chien-Ning Hsu from Kaohsiung Municipal Ta-Tung Hospital with 53 publications (0.98%) (Table 3). Among the top 10 most productive authors, the highest average citations were achieved by Dinan, Timothy G. and Cryan, John F. The H-index, a comprehensive metric evaluating the quantity and impact of scholarly output, was highest for Collado, Maria Carmen and Cryan, John F.

**Table 3:** Top 10 most productive authors in “gut microbiota during pregnancy” research.

Rank	Author	Institution	Countries/regions	Representative papers	Publications *n* (%)	Total citations	Average citations	H-index
Web of Science Core Collection database								
1	Collado, Maria Carmen	Instituto de Agroquímica y Tecnología de Alimentos (IATA-CSIC)	Spain	Mother-to-Infant Microbial Transmission from Different Body Sites Shapes the Developing Infant Gut Microbiome	75 (1.38)	8,322	110.96	37
2	Tain, You-Lin	Kaohsiung Chang Gung Memorial Hospital	China (Taiwan)	Hypertension Programmed by Perinatal High-Fat Diet: Effect of Maternal Gut Microbiota-Targeted Therapy	61 (1.12)	1,546	25.34	24
3	Hsu, Chien-Ning	Kaohsiung Municipal Ta-Tung Hospital Kaohsiung Medical University	China (Taiwan)	Targeting on Gut Microbial Metabolite Trimethylamine-N-Oxide and Short-Chain Fatty Acid to Prevent Maternal High-Fructose-Diet-Induced Developmental Programming of Hypertension in Adult Male Offspring	53 (0.98)	1,276	24.08	22
4	Isolauri, Erika	University of Turku	Finland	Human gut colonization may be initiated in utero by distinct microbial communities in the placenta and amniotic fluid	51 (0.94)	8,186	160.51	33
5	Cryan, John F.	University College Cork	Ireland	The Microbiome-Gut-Brain Axis in Health and Disease	50 (0.92)	12,093	241.86	37
6	Salminen, Seppo	University of Turku	Finland	Maternal gut and breast milk microbiota affect infant gut antibiotic resistome and mobile genetic elements	48 (0.88)	7,797	162.44	32
7	Kozyrskyj, Anita L.	University of Alberta	Canada	Roles of Birth Mode and Infant Gut Microbiota in Intergenerational Transmission of Overweight and Obesity From Mother to Offspring	38 (0.7)	3,025	79.61	25
8	Dinan, Timothy G.	University College Cork	Ireland	The Microbiome-Gut-Brain Axis in Health and Disease	36 (0.66)	10,940	303.89	32
9	Stanton, Catherine	University College Cork	Ireland	Evolution of gut microbiota composition from birth to 24 weeks in the INFANTMET Cohort	36 (0.66)	3,318	92.17	24
10	Hou, Chih-Yao	National Kaohsiung University of Science and Technology	China (Taiwan)	Maternal Garlic Oil Supplementation Prevents High-Fat Diet-Induced Hypertension in Adult Rat Offspring: Implications of H2S-Generating Pathway in the Gut and Kidneys	32 (0.59)	895	27.97	19
Scopus database								
1	Tain, You-Lin	Kaohsiung Chang Gung Memorial Hospital	China (Taiwan)	Hypertension Programmed by Perinatal High-Fat Diet: Effect of Maternal Gut Microbiota-Targeted Therapy	58 (1.05)	1,505	25.95	23
2	Collado, Maria Carmen	Instituto de Agroquímica y Tecnología de Alimentos (IATA-CSIC)	Spain	Mother-to-Infant Microbial Transmission from Different Body Sites Shapes the Developing Infant Gut Microbiome	53 (0.96)	5,355	101.04	27
3	Hsu, Chien-Ning	Kaohsiung Municipal Ta-Tung Hospital Kaohsiung Medical University	China (Taiwan)	Targeting on Gut Microbial Metabolite Trimethylamine-N-Oxide and Short-Chain Fatty Acid to Prevent Maternal High-Fructose-Diet-Induced Developmental Programming of Hypertension in Adult Male Offspring	51 (0.92)	1,222	23.96	21
4	Isolauri, Erika	University of Turku	Finland	Human gut colonization may be initiated in utero by distinct microbial communities in the placenta and amniotic fluid	42 (0.76)	6,042	143.86	27
5	Stanton, Catherine	University College Cork	Ireland	Evolution of gut microbiota composition from birth to 24 weeks in the INFANTMET Cohort	38 (0.69)	4,514	118.79	26
6	Salminen, Seppo	University of Turku	Finland	Maternal gut and breast milk microbiota affect infant gut antibiotic resistome and mobile genetic elements	33 (0.6)	5,651	171.24	26
7	Hou, Chih-Yao	National Kaohsiung University of Science and Technology	China (Taiwan)	Maternal Garlic Oil Supplementation Prevents High-Fat Diet-Induced Hypertension in Adult Rat Offspring: Implications of H2S-Generating Pathway in the Gut and Kidneys	33 (0.6)	983	29.79	20
8	Ross Paul	Universite Paris Saclay	France	The composition of the gut microbiota throughout life, with an emphasis on early life	30 (0.54)	2,959	98.63	21
9	Cryan, John F.	University College Cork	Ireland	The Microbiome-Gut-Brain Axis in Health and Disease	26 (0.47)	3,698	142.23	19
10	Nitert, Marloes Dekker	The University of Queensland	Australia	Increased Systolic and Diastolic Blood Pressure Is Associated With Altered Gut Microbiota Composition and Butyrate Production in Early Pregnancy	25 (0.45)	1880	75.2	16

Results from the Scopus database showed partial overlap with WoSCC but with some differences: Tain, You-Lin (58 publications, 1,505 total citations) and Collado, Maria Carmen (53 publications, 5,355 total citations) remained the top two, but Cryan, John F. dropped to ninth place (26 publications, 3,698 total citations), reflecting differences in database coverage. Notably, Ross Paul from Université Paris Saclay (30 publications, 2,959 total citations) and Nitert, Marloes Dekker from the University of Queensland (25 publications, 1,880 total citations) appeared in the top 10 only in Scopus, highlighting the database’s broader coverage of European and Australasian institutions.

A cross-database comparison showed that authors such as Collado, Maria Carmen; Tain, You-Lin; Isolauri, Erika; and Stanton, Catherine consistently ranked in the top 10 across both databases, confirming the stability and international influence of their research output. At the institutional level, the University of Turku, University College Cork, and Kaohsiung Chang Gung Memorial Hospital formed multi-institutional high-productivity clusters. High H-index and average citation values (e.g., Cryan, John F.) further validated the central role of prolific authors in advancing the field. These findings provide data support for identifying key scholars and optimizing international collaboration networks, while also highlighting the need to consider database differences when assessing author influence.

**Figure 5:**
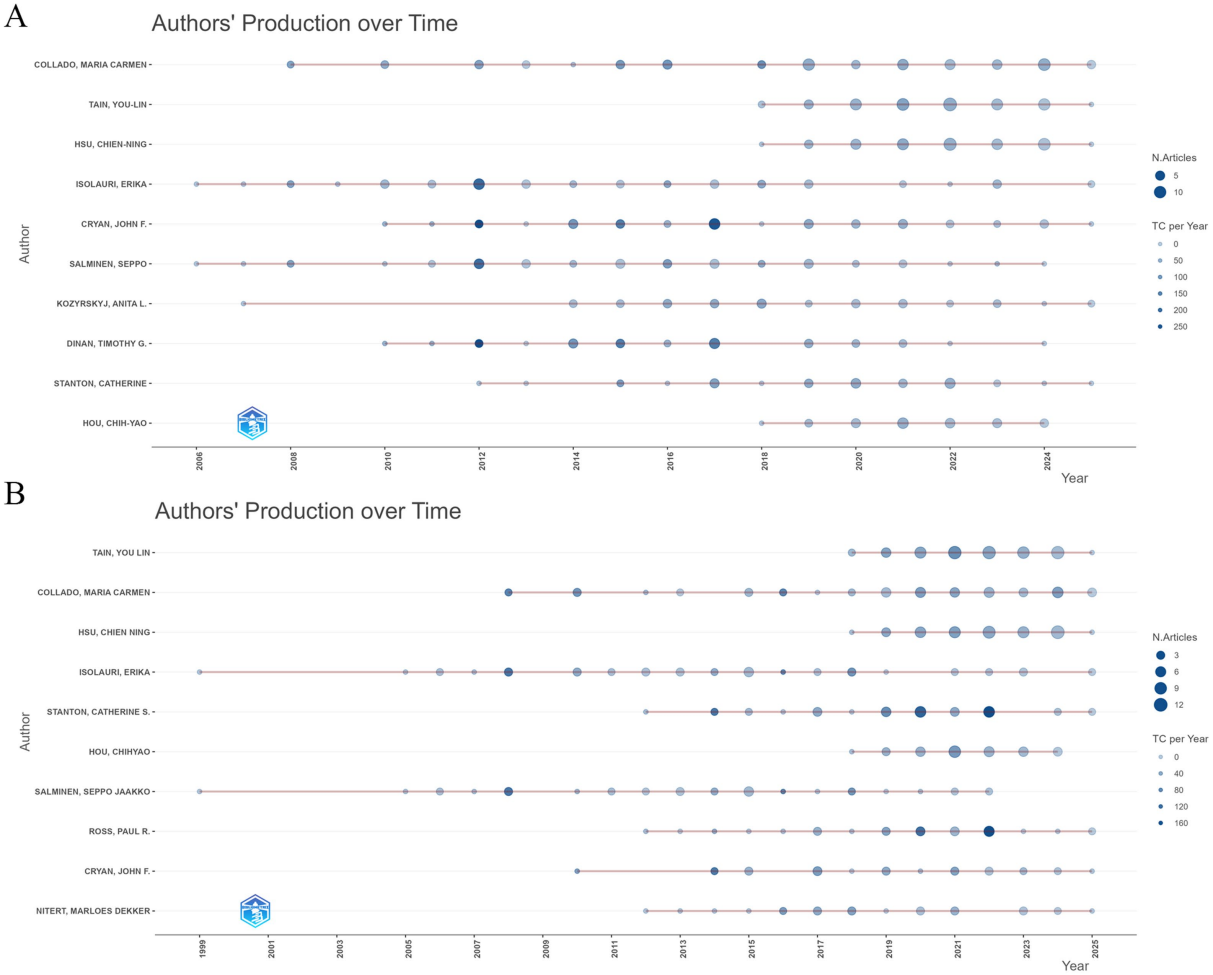
Temporal publication patterns of leading authors: **(A)** WoSCC database; **(B)** Scopus database.

Further analysis of the annual publication output of authors aimed to explore changes in research productivity over time and identify authors who were particularly active during specific periods. Results from the WoSCC (Figure 5A) and Scopus (Figure 5B) databases showed that Isolauri, Erika; Salminen, Seppo; Kozyrskyj, Anita L.; and Collado, Maria Carmen have consistently published research in the “gut microbiota during pregnancy” field over the past three decades, demonstrating their long-standing research foundation and sustained interest. In contrast, Tain, You-Lin; Hsu, Chien-Ning; and Hou, Chih-Yao have significantly increased their research activity in recent years, suggesting important advancements in specific research directions.

### 3.4 Discipline and journal distribution

#### 3.4.1 Disciplines

In terms of disciplinary publication, as shown in Table 4, based on the WoSCC database, Microbiology (16.99%) was the most concentrated discipline, aligning with the field’s focus on microbiota composition, function, and dynamics. Nutrition (13.44%) and Immunology (9.19%) ranked second and third, respectively, reflecting the emphasis on dietary interventions, metabolic regulation, and maternal–infant immune adaptation. Notably, Multidisciplinary Sciences (6.44%) appeared in the top 10, confirming the trend toward interdisciplinary integration. In the Scopus database, Medicine (58.88%) dominated, followed by Immunology and Microbiology (23.57%) and Biochemistry, Genetics, and Molecular Biology (22.45%).

**Table 4:** Top 10 disciplinary categories in the field of “gut microbiota during pregnancy” research.

Rank	Web of science categories	Publications *n* (%)	Scopus subject area	Publications *n* (%)
1	Microbiology	923 (16.99)	Medicine	3,263 (58.88)
2	Nutrition Dietetics	730 (13.44)	Immunology and Microbiology	1,306 (23.57)
3	Immunology	499 (9.19)	Biochemistry, Genetics and Molecular Biology	1,244 (22.45)
4	Pediatrics	443 (8.16)	Agricultural and Biological Sciences	979 (17.67)
5	Biochemistry Molecular Biology	409 (7.53)	Nursing	689 (12.43)
6	Multidisciplinary Sciences	350 (6.44)	Pharmacology, Toxicology and Pharmaceutics	303 (5.47)
7	Gastroenterology Hepatology	297 (5.47)	Multidisciplinary	296 (5.34)
8	Endocrinology Metabolism	282 (5.19)	Neuroscience	277 (5)
9	Neurosciences	260 (4.79)	Environmental Science	262 (4.73)
10	Food Science Technology	241 (4.44)	Chemistry	202 (3.64)

#### 3.4.2 Journal distribution

Analysis of journal sources using the bibliometrix package in R revealed a concentration of research output in this field. As shown in Table 5, the top 10 journals in the WoSCC database contributed over 20% of the publications, forming a core journal group that dominates knowledge dissemination. *Nutrients* led with 244 publications (4.49%), highlighting the key role of nutrition journals in this interdisciplinary field. *Frontiers in Microbiology* (164 publications, 3.02%) and *Scientific Reports* (135 publications, 2.49%) ranked second and third, respectively. Notably, *Plos One*, although ranking fourth with 105 publications (1.93%), showed strong impact with 6,417 total citations and an average of 61.11 citations per article. *Microbiome*, despite having fewer publications (69, 1.27%), had a high average of 89.78 citations per article and the highest journal impact factor (12.7), indicating broad academic recognition of its published research.

**Table 5:** Top 10 journals publishing “gut microbiota during pregnancy” research.

Rank	Journal	Publications *n* (%)	Total citations	Average citations	H-index	JCR category	Category quartile	Journal impact factor (2024)
Web of Science Core Collection database								
1	Nutrients	244 (4.49)	6,390	26.19	40	Nutrition and Dietetics	Q1	5
2	Frontiers in Microbiology	164 (3.02)	4,570	27.87	34	Microbiology	Q1	4.5
3	Scientific Reports	135 (2.49)	5,102	37.79	39	Multidisciplinary Sciences	Q1	3.9
4	Plos One	105 (1.93)	6,417	61.11	42	Multidisciplinary Sciences	Q2	2.6
5	Gut Microbes	104 (1.91)	3,914	37.63	35	Microbiology	Q1	11
6	International Journal of Molecular Sciences	90 (1.66)	2,145	23.83	27	Biochemistry and Molecular Biology	Q1	4.9
7	Microorganisms	79 (1.45)	3,283	41.56	18	Microbiology	Q2	4.2
8	Frontiers in Cellular and Infection Microbiology	75 (1.38)	1,665	22.2	23	Microbiology	Q1	4.8
9	Frontiers in Immunology	69 (1.27)	3,513	50.91	28	Immunology	Q1	5.9
10	Microbiome	69 (1.27)	6,172	89.45	39	Microbiology	Q1	12.7
Scopus database								
1	Nutrients	257 (4.64)	9,017		48	Nutrition and Dietetics	Q1	5
2	Frontiers in Microbiology	176 (3.18)	4,621		35	Microbiology	Q1	4.5
3	Gut Microbes	140 (2.53)	5,714		41	Microbiology	Q1	11
4	Scientific Reports	115 (2.08)	5,220		38	Multidisciplinary Sciences	Q1	3.9
5	Plos One	102 (1.84)	6,159		43	Multidisciplinary Sciences	Q2	2.6
6	Frontiers in Cellular and Infection Microbiology	98 (1.77)	2,813		27	Microbiology	Q1	4.8
7	International Journal of Molecular Sciences	85 (1.53)	2003		27	Biochemistry and Molecular Biology	Q1	4.9
8	Microbiome	77 (1.39)	7,101		39	Microbiology	Q1	12.7
9	Microorganisms	65 (1.17)	3,458		17	Microbiology	Q2	4.2
10	Frontiers in Immunology	62 (1.12)	3,165		27	Microbiology	Q1	4.5

Results from the Scopus database were highly consistent with WoSCC. The top 10 journals were distributed in JCR Q1/Q2 quartiles, covering microbiology, nutrition, immunology, and multidisciplinary sciences, fully affirming the interdisciplinary nature of research on gut microbiota during pregnancy.

#### 3.4.3 Top 10 highly cited references and co-cited references

**Figure 6:**
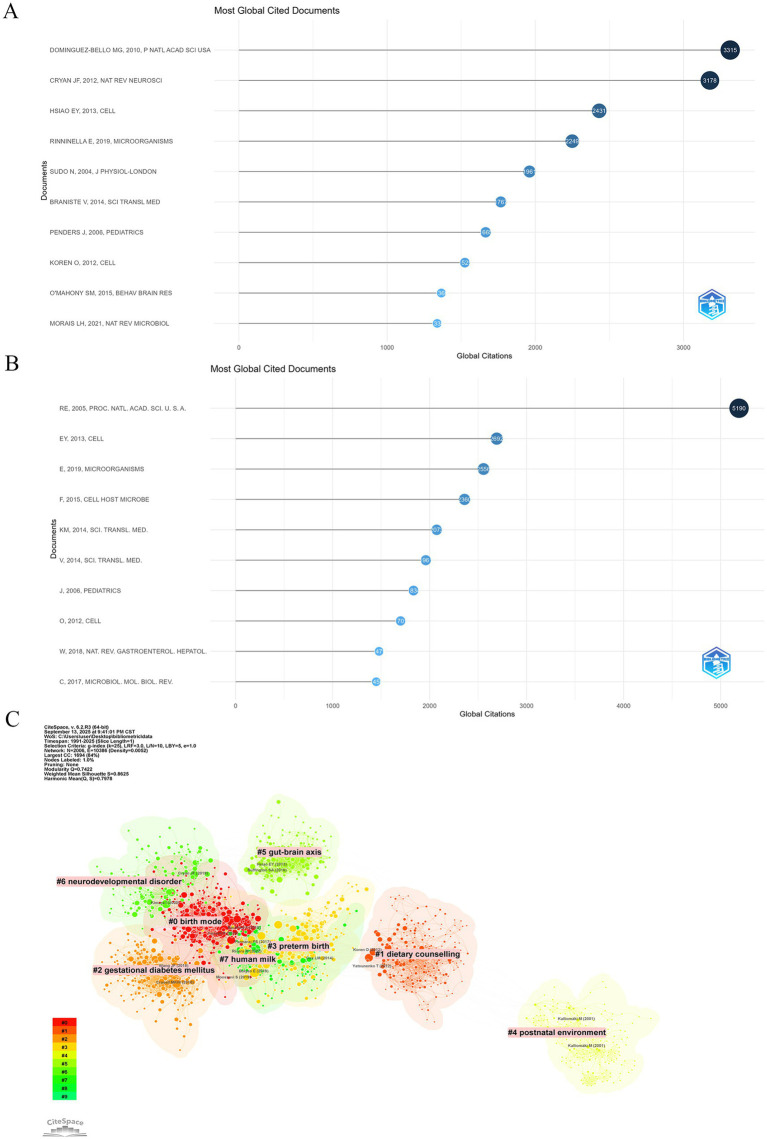
Citation analysis of core reference. **(A)** Top 10 most cited references in WoSCC; **(B)** Top 10 most cited references in Scopus; **(C)** Co-citation reference clusters in WoSCC.

In-depth analysis of cited references and co-cited references was conducted using the bibliometrix package in R. The top 10 highly cited articles in the “gut microbiota during pregnancy” field showed consistency between the WoSCC (Figure 6A) and Scopus (Figure 6B) databases. Five articles appeared in the top 10 of both databases (Table 6), forming the knowledge foundation of the field. These included: Hsiao et al. (2013) on microbiota modulation of neurodevelopmental abnormalities; Rinninella et al. (2019) reviewing the definition of a healthy gut microbiota; Penders et al. (2006) exploring factors influencing infant gut microbiota; Koren et al. (2012) on host-microbiome interactions during pregnancy; and Braniste et al. (2014) demonstrating microbiota influence on blood–brain barrier permeability. The shared highly cited articles focused on early-life microbiota colonization, the gut-brain axis, and host-microbiome interactions during pregnancy, highlighting the foundational importance of these directions.

**Table 6:** Top 10 most cited references in “gut microbiota during pregnancy” research.

Rank	Author	Title	Journal	DOI	Year of publication	Total citations	Normalized global citations
Web of Science Core Collection database							
1	Dominguez-Bello, Maria Gloria	Delivery mode shapes the acquisition and structure of the initial microbiota across multiple body habitats in newborns	Proceedings of The National Academy of Sciences of The United States of America	10.1073/pnas.1002601107	2010	3,315	15.74
2	Cryan, John F.	Mind-altering microorganisms: the impact of the gut microbiota on brain and behavior	Nature Reviews Neuroscience	10.1038/nrn3346	2012	3,178	15.64
3	Hsiao, Elaine	Microbiota Modulate Behavioral and Physiological Abnormalities Associated with Neurodevelopmental Disorders	Cell	10.1016/j.cell.2013.11.024	2013	2,431	20.87
4	Rinninella, Emanuele	What is the Healthy Gut Microbiota Composition? A Changing Ecosystem across Age, Environment, Diet, and Diseases	Microorganisms	10.3390/microorganisms7010014	2019	2,249	37.76
5	Sudo, Nobuyuki	Postnatal microbial colonization programs the hypothalamic–pituitary–adrenal system for stress response in mice	Journal of Physiology-London	10.1113/jphysiol.2004.063388	2004	1961	9.59
6	Braniste, Viorica	The gut microbiota influences blood–brain barrier permeability in mice	Science Translational Medicine	10.1126/scitranslmed.3009759	2014	1767	13.72
7	Penders, John	Factors influencing the composition of the intestinal microbiota in early infancy	Pediatrics	10.1542/peds.2005-2824	2006	1,665	9.36
8	Koren, Omry	Host Remodeling of the Gut Microbiome and Metabolic Changes during Pregnancy	Cell	10.1016/j.cell.2012.07.008	2012	1,525	7.51
9	O’Mahony, Siobhain M.	Serotonin, tryptophan metabolism and the brain-gut-microbiome axis	Behavioral Brain Research	10.1016/j.bbr.2014.07.027	2015	1,366	11.09
10	Morais, Livia	The gut microbiota-brain axis in behavior and brain disorders	Nature Reviews Microbiology	10.1038/s41579-020-00460-0	2021	1,338	42.81
Scopus database							
1	Ley, Ruth	Obesity alters gut microbial ecology	Proceedings of The National Academy of Sciences of The United States of America	10.1073/pnas.0504978102	2005	5,190	9.04
2	Hsiao, Elaine	Microbiota Modulate Behavioral and Physiological Abnormalities Associated with Neurodevelopmental Disorders	Cell	10.1016/j.cell.2013.11.024	2013	2,692	20.08
3	Rinninella, Emanuele	What is the Healthy Gut Microbiota Composition? A Changing Ecosystem across Age, Environment, Diet, and Diseases	Microorganisms	10.3390/microorganisms7010014	2019	2,556	37.27
4	Bäckhed, Fredrik	Dynamics and Stabilization of the Human Gut Microbiome during the First Year of Life	Cell Host and Microbe	10.1016/j.chom.2015.05.012	2015	2,360	21.10
5	Aagaard, Kjersti	The placenta harbors a unique microbiome	Science Translational Medicine	10.1126/scitranslmed.3008599	2014	2073	11.62
6	Braniste, Viorica	The gut microbiota influences blood–brain barrier permeability in mice	Science Translational Medicine	10.1126/scitranslmed.3009759	2014	1961	10.99
7	Penders, John	Factors influencing the composition of the intestinal microbiota in early infancy	Pediatrics	10.1542/peds.2005-2824	2006	1834	11.24
8	Koren, Omry	Host Remodeling of the Gut Microbiome and Metabolic Changes during Pregnancy	Cell	10.1016/j.cell.2012.07.008	2012	1701	13.03
9	Bäckhed, Fredrik	Bile acid-microbiota crosstalk in gastrointestinal inflammation and carcinogenesis	Nature Reviews Gastroenterology and Hepatology	10.1038/nrgastro.2017.119	2018	1,479	17.20
10	Milani, Christian	The First Microbial Colonizers of the Human Gut: Composition, Activities, and Health Implications of the Infant Gut Microbiota	Microbiology and molecular biology reviews	10.1128/MMBR.00036-17	2017	1,450	14.65

Analysis of highly co-cited references revealed the core knowledge base and key methodological frameworks in the field. The top 10 most frequently co-cited references are listed in Table 7. Four foundational studies appeared prominently in both databases: (1) Koren et al. (2012) on host and microbiome remodeling during pregnancy, ranked first in WoSCC, establishing pregnancy as a key driver of microbial succession; (2) Dominguez-Bello et al. (2010) on the impact of delivery mode on neonatal microbiota colonization; (3) Backhed et al. (2015) on the longitudinal dynamics and stabilization of the infant gut microbiome in the first year of life; and (4) Aagaard et al. (2014) proposing the concept of a placental microbiome, which, despite controversy, demonstrated significant influence through its co-citation strength. Additionally, two key methodological papers were included: Caporaso et al. (2010) on the QIIME pipeline and Callahan et al. (2016) on the DADA2 analysis pipeline, highlighting the indispensable role of high-throughput sequencing data analysis methods. Yatsunenko et al. (2012) on human gut microbiome variations across age and geography was also co-cited, providing an important comparative framework for subsequent research.

**Table 7:** Top 10 most co-cited references in “gut microbiota during pregnancy” research.

Rank	Author	Title	Journal	DOI	Year of publication	Number of references co-cited
Web of Science Core Collection database						
1	Koren, Omry	Host remodeling of the gut microbiome and metabolic changes during pregnancy	Cell	10.1016/j.cell.2012.07.008	2012	790
2	Dominguez-Bello, Maria Gloria	Delivery mode shapes the acquisition and structure of the initial microbiota across multiple body habitats in newborns	Proceedings of The National Academy of Sciences of The United States of America	10.1073/pnas.1002601107	2010	621
3	Bäckhed, Fredrik	Dynamics and Stabilization of the Human Gut Microbiome during the First Year of Life	Cell Host and Microbe	10.1016/j.chom.2015.04.004	2015	552
4	Aagaard, Kjersti	The placenta harbors a unique microbiome	Science Translational Medicine	10.1126/scitranslmed.3008599	2014	447
5	Caporaso, J Gregory	QIIME allows analysis of high-throughput community sequencing data	Nature Methods	10.1038/nmeth.f.303	2010	432
6	Yatsunenko, Tanya	Human gut microbiome viewed across age and geography	Nature	10.1038/nature11053	2012	429
7	Turnbaugh, Peter James	An obesity-associated gut microbiome with increased capacity for energy harvest	Nature	10.1038/nature05414	2006	371
8	Callahan, Benjamin John	DADA2: High-resolution sample inference from Illumina amplicon data	Nature Methods	10.1038/NMETH.3869	2016	364
9	Penders, John	Factors influencing the composition of the intestinal microbiota in early infancy	Pediatrics	10.1542/peds.2005-2824	2006	337
10	Ferretti, Pamela	Mother-to-Infant Microbial Transmission from Different Body Sites Shapes the Developing Infant Gut Microbiome	Cell Host and Microbe	10.1016/j.chom.2018.06.005	2018	322
Scopus database						
1	Bäckhed, Fredrik	Dynamics and Stabilization of the Human Gut Microbiome during the First Year of Life	Cell Host and Microbe	10.1016/j.chom.2015.04.004	2015	205
2	Aagaard, Kjersti	The placenta harbors a unique microbiome	Science Translational Medicine	10.1126/scitranslmed.3008599	2014	178
3	Koren, Omry	Host remodeling of the gut microbiome and metabolic changes during pregnancy	Cell	10.1016/j.cell.2012.07.008	2012	154
4	Dominguez-Bello, Maria Gloria	Delivery mode shapes the acquisition and structure of the initial microbiota across multiple body habitats in newborns	Proceedings of The National Academy of Sciences of The United States of America	10.1073/pnas.1002601107	2010	141
5	Caporaso, J Gregory	QIIME allows analysis of high-throughput community sequencing data	Nature Methods	10.1038/nmeth.f.303	2010	106
6	Yatsunenko, Tanya	Human gut microbiome viewed across age and geography	Nature	10.1038/nature11053	2012	94
7	Milani, Christian	The first microbial colonizers of the human gut: composition, activities, and health implications of the infant gut microbiota	Microbiology and molecular biology reviews	10.1128/MMBR.00036-17	2017	82
8	Callahan, Benjamin John	DADA2: High-resolution sample inference from Illumina amplicon data	Nature Methods	10.1038/NMETH.3869	2016	73
9	Azad, Meghan B	Gut microbiota of healthy canadian infants: profiles by mode of delivery and infant diet at 4 months	Canadian Medical Association Journal	10.1503/cmaj.121189	2013	72
10	Arrieta, Marie-Claire	Early infancy microbial and metabolic alterations affect risk of childhood asthma	Science Translational Medicine	10.1126/scitranslmed.aab2271	2015	69

To further analyze co-citation relationships, we performed a visualization analysis of co-cited references in the WoSCC database using CiteSpace (Figure 6C). CiteSpace parameters were set as follows: time slicing (1991–2025), years per slice (1), node type (keyword), selection criteria (g-index, *k* = 25), no pruning. Eight major clusters were identified: Birth mode, dietary counseling, GDM, preterm birth, postnatal environment, gut-brain axis, neurodevelopmental disorder, and human milk.

### 3.5 Keyword analysis

Keyword analysis to elucidate the knowledge structure and research hotspots in this field, we employed VOSviewer to perform a co-occurrence network analysis of literature keywords. To ensure clustering accuracy and representativeness, we excluded core search terms and their synonyms, while merging synonymous terms in the database. This step effectively mitigated clustering distortion and centralization bias caused by terminological diversity. In the co-occurrence network, line thickness visually represents the frequency of keyword co-occurrence in the same literature, with thicker lines indicating higher co-occurrence rates.

**Figure 7:**
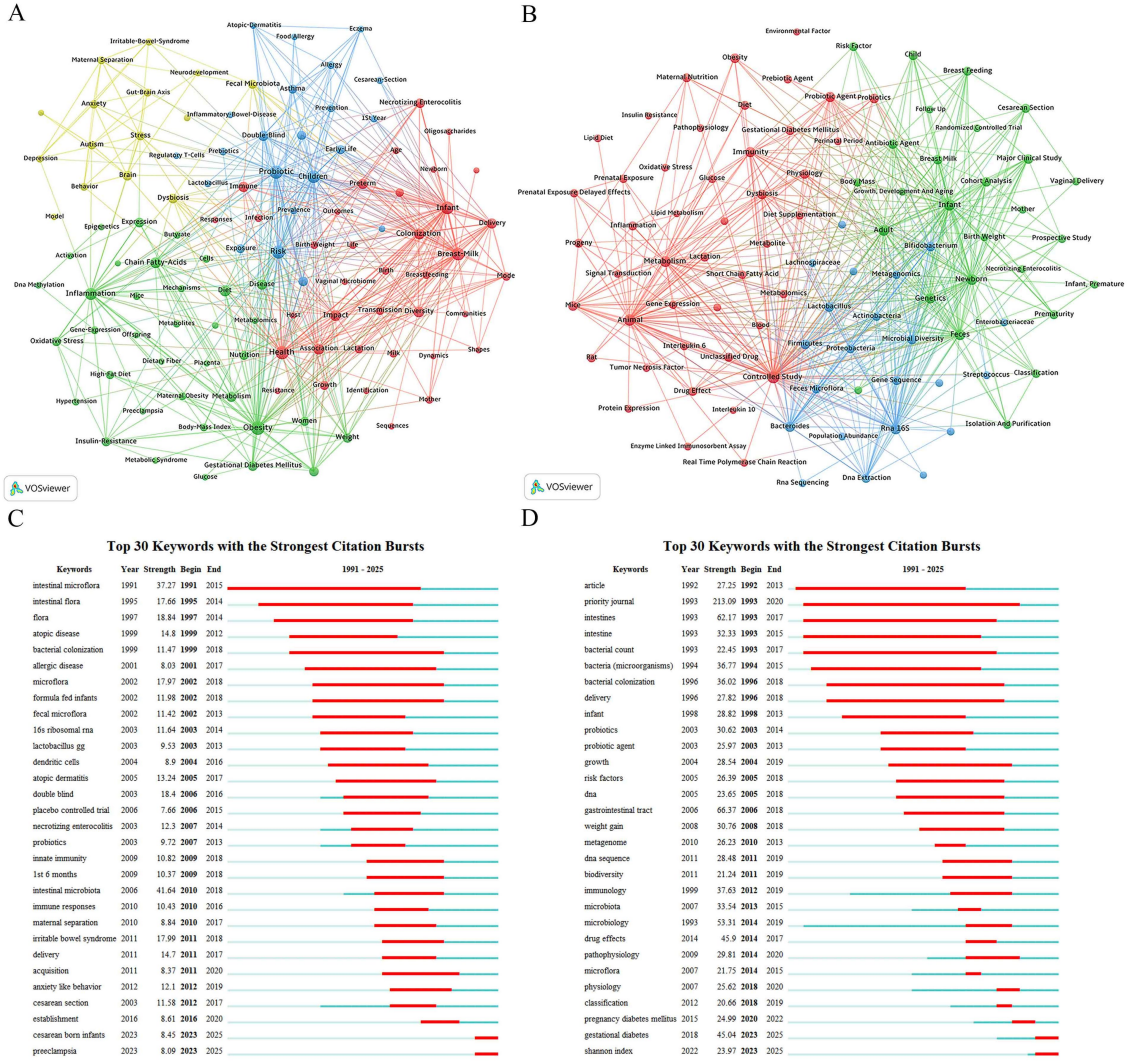
Keyword analysis. Keyword co-occurrence networks in WoSCC database **(A)** and Scopus database **(B)**; keyword burst detection in WoSCC database **(C)** and Scopus database **(D)**.

Results from the WoSCC database (Figure 7A) reveal that “Obesity” (607 occurrences) is the most frequent keyword, underscoring its central role in current research. “Risk” (572 occurrences) and “Probiotic” (555 occurrences) rank second and third. The co-occurrence network is clearly divided into four main clusters, each representing a distinct and concentrated research direction.

The green cluster centers on “Obesity” and focuses on the pathophysiological mechanisms of obesity and metabolic syndrome. It traces a research trajectory from phenotypic markers (e.g., “Overweight,” “Body-Mass Index,” “Gestational Diabetes Mellitus,” “Preeclampsia,” “Hypertension”) to molecular mechanisms, emphasizing inflammation (“Inflammation,” “Oxidative Stress,” “Activation”) and metabolic regulation (“Metabolism,” “Insulin-Resistance,” “Glucose”). Studies investigate how dietary factors (“Diet,” “High-Fat Diet,” “Dietary Fiber”) influence gut microbiota metabolites (“Short-Chain Fatty Acids,” “Butyrate,” “Metabolites”), triggering host epigenetic changes (“Epigenetics,” “DNA Methylation”) and gene expression alterations (“Gene-Expression,” “Expression”), ultimately impacting maternal-fetal health (“Maternal Obesity,” “Maternal Diet,” “Offspring,” “Placenta”). This cluster integrates multi-level evidence from animal models (“Mice,” “Cells”) to human studies, unraveling the complex network of obesity-related metabolic diseases.

The blue cluster, led by “Risk” and “Probiotic,” addresses early-life microbiota interventions and their long-term health outcomes, particularly allergic and immune diseases. This direction is characterized by clinical intervention and epidemiological features, as evidenced by keywords like “Double-Blind,” “Supplementation,” “Meta-analysis,” and “Prevalence.” Research focuses on early-life interventions (“Early-Life,” “1st Year,” “Children”) using probiotics (“Probiotic,” “Lactobacillus”), prebiotics (“Prebiotics”), or avoiding disruptors like antibiotics (“Antibiotics”) and cesarean section (CS) (“Cesarean-Section”) to modulate the immune system (“Regulatory T-Cells”) and prevent diseases such as “Asthma,” “Allergy,” “Atopic-Dermatitis,” “Eczema,” “Food Allergy,” and “Inflammatory-Bowel-Disease.”

The red cluster revolves around “Health,” “Infant,” and “Breast-Milk,” emphasizing the establishment and transmission of maternal–infant microbiota and its impact on infant health. Research threads begin with delivery and feeding modes (“Delivery,” “Mode,” “Breastfeeding,” “Lactation”), exploring their effects on neonatal gut microbiota colonization (“Colonization”) and diversity (“Diversity”). Studies link early microbiota establishment to infant growth (“Growth,” “Birth-Weight”), immune responses (“Immune,” “Responses”), and disease risks (“Necrotizing Enterocolitis,” “Infection”). A highlight is the in-depth investigation of breast milk components (“Breast-Milk,” “Milk,” “Oligosaccharides,” “Human-Milk Oligosaccharides”), revealing mechanisms by which breast milk shapes neonatal gut microbiota (“Bifidobacteria”) to promote health. Keywords like “Sequences” and “Identification” reflect heavy reliance on high-throughput sequencing technologies.

The smaller yellow cluster, centered on “Dysbiosis” and “Brain,” represents the rapidly growing “gut-brain axis” field. It explores links between gut microbiota and central nervous system function, as well as psychiatric and behavioral disorders. Research spans clinical conditions (“Autism,” “Depression,” “Anxiety,” “Irritable-Bowel-Syndrome”) and animal models (“Model,” “Maternal Separation,” “Maternal Immune Activation,” “Prenatal Stress”). Keywords like “Stress,” “Behavior,” and “Neurodevelopment” highlight the focus: early-life (including prenatal) stress and immune activation induce dysbiosis, impacting brain development and behavior, potentially leading to neuropsychiatric disorders.

To validate and comprehensively examine the knowledge structure of the research field, this study further conducted a keyword co-occurrence analysis on the literature in the Scopus database. As shown in Figure 7B, its network structure shares commonalities with that of the WoSCC database while also exhibiting unique emphases.

In the Scopus database, “Controlled Study” (2,116 occurrences) emerged as the most frequently mentioned keyword. It was followed by “Animal” (1,944 occurrences) and “Infant” (1,778 occurrences). Through cluster analysis, the keywords were divided into three clusters.

The red cluster, the largest and most content-rich, was dominated by “Controlled Study” and “Animal,” clearly pointing to mechanism-based explorations through animal experiments. This direction delved into the role of “Dysbiosis” under specific pathophysiological conditions, with core models including “Obesity,” “Gestational Diabetes Mellitus,” and “Inflammation.” Research utilized interventions such as “Probiotic Agent,” “Prebiotic Agent,” “Diet Supplementation,” “Fecal Microbiota Transplantation,” and “High-Fat Diet” to observe their effects on the “Metabolism” and “Immunity” of “Progeny” or adult individuals. Mechanistic studies extended to the molecular level, employing techniques like “Real Time Polymerase Chain Reaction,” “Enzyme Linked Immunosorbent Assay,” and “Metabolomics” to investigate changes in “Gene Expression,” “Protein Expression,” “Signal Transduction,” and “Metabolites” such as “Short Chain Fatty Acid,” as well as the roles of key inflammatory factors like “Interleukin 6” and “Tumor Necrosis Factor.” This cluster systematically revealed how interventions from “Prenatal Exposure” to the “Perinatal Period” influence host pathophysiology through molecular pathways.

The green cluster, centered around “Infant,” “Newborn,” and “Adult,” primarily represented population-based clinical and translational medical research. Its research focus centered on the early life stages, encompassing the growth and development process from “Prematurity” and “Birth Weight” to “Child” (“Growth, Development and Aging”). Classic clinical research methods such as “Cohort Analysis,” “Prospective Study,” and “Randomized Controlled Trial” were employed to explore key exposure factors affecting infant health, including “Cesarean Section,” “Vaginal Delivery,” “Breast Feeding,” and the use of “Antibiotic Agent.” Health outcomes were closely tied to specific clinical issues like “Necrotizing Enterocolitis” and “Body Mass,” with microbiome analysis conducted through “Feces” samples. This cluster overall embodied a longitudinal observational research paradigm from “Mother” to “Progeny.”

The blue cluster featured distinct methodological characteristics, with “RNA 16S” (16S rRNA gene sequencing) and “Metagenomics” at its core. This cluster encompassed the key technical processes of gut microbiome research, from “Dna Extraction” to “Polymerase Chain Reaction,” then to “High Throughput Sequencing” and “RNA Sequencing,” culminating in “Population Abundance” analysis and “Classification.” Correspondingly, the keywords in this cluster also listed in detail the core gut microbial phyla and genera identified through these methods, such as “Bacteroides,” “Bifidobacterium,” “Lactobacillus,” “Firmicutes,” and “Proteobacteria.” This cluster essentially served as the technical and analytical foundation supporting the entire research field, providing data sources and research tools for the other two clusters.

**Figure 8:**
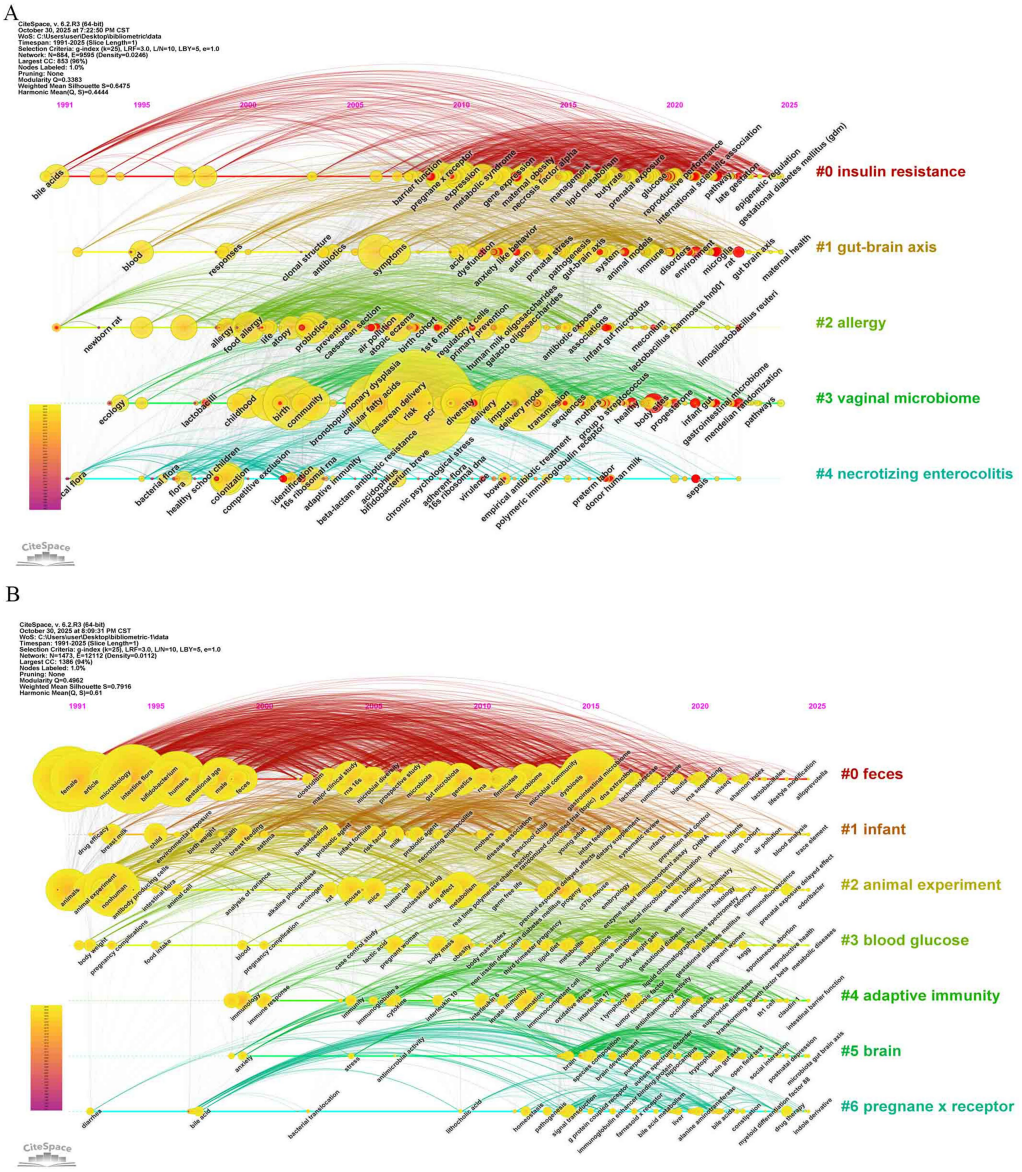
Timeline view of keywords. **(A)** WoSCC database; **(B)** Scopus database.

To identify research hotspots with high burst strength and distinct temporal characteristics in the “gut microbiota during pregnancy” field, this study detected the top 30 keywords with the strongest citation bursts in the WoSCC and Scopus databases using CiteSpace (Figures 7C,D) and generated a timeline view of the field over the past 35 years (Figures 8A,B) to reveal thematic evolution and development dynamics. CiteSpace parameters were set as follows: time slicing (1991–2025), years per slice (1), node type (keyword), selection criteria (g-index, *k* = 25), no pruning.

The two databases revealed differentiated characteristics of the research frontiers. The WoSCC database timeline viewer showed the top five clusters as insulin resistance, gut-brain axis, allergy, vaginal microbiome, and necrotizing enterocolitis (Figure 8A). Keywords clearly displayed a three-stage evolutionary path from descriptive research to mechanistic and systemic studies (Figure 7C): the early stage (before 2010) was dominated by terms such as “intestinal microflora,” “flora,” and “atopic disease,” reflecting the initial focus on microbial composition and its association with allergic diseases. Between 2010 and 2018, keywords such as “irritable bowel syndrome,” and “anxiety like behavior” indicated a shift toward mechanistic exploration, particularly focusing on the gut-brain axis and immune regulation mechanisms. Recently (2023–2025), the emergence of “cesarean born infants” and “preeclampsia” showed an extension of research to delivery modes and associations with pregnancy complications.

In contrast, the Scopus database exhibited a more detailed cluster structure (Figure 8B), with the top seven clusters being feces; infant; animal experiment; blood glucose; adaptive immunity; brain; pregnane x receptor. The burst word list in Scopus included more general medical or methodological keywords (Figure 7D), such as “article,” “priority journal” and “drug effects,” which had high burst strength but weaker direct research relevance. Nevertheless, Scopus also captured biologically significant keywords with strong bursts, such as “bacterial colonization,” “probiotics,” and “gastrointestinal tract.” Notably, “gestational diabetes” and “Shannon index” showed high burst strength between 2023 and 2025, indicating current research frontiers focused on the association between gestational diabetes and microbiota, as well as quantitative analysis of microbial diversity.

In summary, both databases indicate that the field has moved beyond mere description of microbiota composition toward exploring underlying mechanisms and multi-omics integration. Mechanistic research on pregnancy complications, especially GDM and preeclampsia, constitutes the current clinical frontier. The microbiota-gut-brain axis and its impact on maternal and infant neuropsychiatric health (e.g., anxiety, depression, autism spectrum disorder) form another key direction of shared interest.

## 4 Discussion

This study provides a systematic bibliometric analysis of the global research landscape concerning the gut microbiota during pregnancy, utilizing a dual-database approach incorporating the WoSCC and Scopus. The primary methodological contribution of this study is the comparative analysis enabled by this dual-strategy. Recognizing that different bibliographic databases have distinct coverage policies and inherent biases, the use of both WoSCC and Scopus was intended to mitigate the risk of a skewed or incomplete representation of the field. WoSCC is often recognized for its selective journal inclusion, particularly of high-impact English-language publications, which can lend a certain prestige to its indexed content. In contrast, Scopus offers broader coverage, including a more extensive array of journals from regions like Europe and the Asia-Pacific. This difference was observable in our results; for instance, China’s publication output appeared more prominent in Scopus, while the United States maintained a leadership position in citation-based impact metrics within WoSCC. Therefore, this approach allows for a more comprehensive and balanced mapping of the academic terrain than would be feasible with a single database, constructing a more robust and three-dimensional global perspective on the field’s development.

The analysis reveals that research on the gut microbiota during pregnancy has experienced a phase of exponential growth since approximately 2014, with annual publication output showing a consistent upward trajectory. This trend reflects a widespread and deepening academic recognition of the critical role played by maternal microbial communities in shaping both immediate and long-term maternal and infant health outcomes, solidifying this area as a globally significant frontier in biomedical science. The rapid maturation of this field is intrinsically linked to the deep integration of multidisciplinary methodologies. Advancements in high-throughput sequencing technologies, the refinement of metagenomic analysis pipelines (Asnicar et al., 2017; Liu et al., 2023; Vaishampayan et al., 2010; van de Wijgert et al., 2020), and the widespread application of sophisticated bioinformatics tools (Caporaso et al., 2010; Callahan et al., 2016) have been fundamental drivers, a fact corroborated by both keyword burst analysis and the content of highly co-cited references. Concurrently, the cross-disciplinary fusion of insights from nutrition, immunology, microbiology, and neuroscience has progressively shifted the research perspective from initial descriptions of microbial composition toward a more mechanistic exploration of microbiota-host interactions, metabolic pathway regulation, and ultimate clinical translation. The significant representation of microbiology, nutrition, and immunology in the disciplinary distribution, alongside the prominent role of comprehensive journals (e.g., *PLOS ONE*, *Scientific Reports*) and high-impact specialized journals (e.g., *Microbiome*, *Gut Microbes*) as primary publication venues, collectively confirms the highly interdisciplinary nature of this scientific endeavor.

### 4.1 Synthesis of research hotspots and evolving themes

A synthesis of the bibliometric data, including keyword co-occurrence, citation analysis, and the review of seminal publications, allows for the identification of several dominant and interconnected research hotspots that define the current intellectual structure of the field.

#### 4.1.1 Maternal metabolic health and gestational complications

A primary research cluster focuses on the intricate interplay between the maternal gut microbiome and metabolic health during gestation, particularly in the context of specific pregnancy complications. Core themes within this cluster include obesity, GDM, insulin resistance, and preeclampsia. Research has consistently demonstrated that maternal pre-pregnancy overweight/obesity and excessive gestational weight gain are associated with a state of microbial dysbiosis, characterized by reduced microbial diversity, an elevated *Firmicutes/Bacteroidetes* ratio, and taxonomic shifts such as a decreased abundance of beneficial *Bifidobacterium and Akkermansia* alongside an increase in pro-inflammatory microbes (Houttu et al., 2018; Zacarias et al., 2018). In GDM, this dysbiosis is often more pronounced, synergistically exacerbating metabolic dysfunction and systemic inflammation (Li J. et al., 2024; Mei et al., 2025). The investigation into underlying mechanisms is heavily centered on host-microbiota crosstalk, with a strong emphasis on microbial metabolites and metabolomics. Short-chain fatty acids (SCFAs) have emerged as central regulators. For instance, in high-fat-diet-induced GDM models, reduced SCFA production has been shown to activate intestinal CD36-mediated lipid absorption via the HDAC3-H3K27ac-PPARγ axis, thereby promoting systemic insulin resistance (Chen et al., 2024). Human studies corroborate these findings, showing that women with GDM exhibit decreased circulating levels of acetate, propionate, and butyrate, which correlate with altered expression of SCFA receptors (GPR41/43) and metabolic markers in placental tissue (Wang et al., 2022), affirming SCFAs as pivotal modulators of the gut-islet axis in pregnancy. Nutrition is recognized as a key modulator linking the microbiota to these metabolic outcomes. Different dietary patterns elicit divergent microbial metabolic functions; for example, high-complex-carbohydrate diets can increase the abundance of beneficial bacteria and enhance carbohydrate metabolism, whereas regular or high-fat diets may activate pro-inflammatory pathways (Sugino et al., 2024). This research, while focused on maternal health, is ultimately directed toward understanding how optimizing the gestational metabolic environment can confer long-term health benefits to the offspring.

#### 4.1.2 Early-life microbial colonization and determinants

A second major research axis is dedicated to understanding the origins, colonization, and establishment of the gut microbiota in early life and its profound implications for infant health and development. This cluster emphasizes the neonatal period as a critical window for microbial acquisition and assembly. Two major determinants are extensively studied: delivery mode and feeding practices. CS delivery consistently demonstrates profound effects on neonatal microbiota acquisition compared to vaginal delivery. Infants born via CS exhibit reduced microbial diversity, depletion of beneficial genera such as *Bifidobacterium* and *Bacteroides*, and an enrichment of potential pathogens (Kim et al., 2020; Madan et al., 2016; Reznik et al., 2024). These compositional alterations coincide with functional shifts in intestinal metabolism, including modified bile acid oxidation and disrupted carbohydrate and amino acid pathways (Hoen et al., 2021; Wang W. et al., 2020), suggesting a mechanistic link between CS-associated dysbiosis and long-term health risks. The second key factor is feeding mode, with breast milk being regarded not only as a nutritional source but also as a complex vehicle for bioactive molecules and microorganisms crucial for driving microbiota maturation (Chen et al., 2019; Merter and Altay, 2024), immune development (Li Y. et al., 2024; Wang et al., 2024; Zhou et al., 2020), and growth (Ajeeb et al., 2024; Rendina et al., 2019). Even minimal formula supplementation can shift the infant microbiota toward a profile more typical of formula-fed infants, attenuating the protective effects of exclusive breastfeeding (Madan et al., 2016). A significant sub-theme within this cluster concerns the vulnerable population of preterm infants, where aberrant microbial colonization is strongly associated with necrotizing enterocolitis (NEC). Pre-NEC microbial signatures feature increased *Proteobacteria* (especially *Gammaproteobacteria* and *Enterobacteriaceae*) and decreased beneficial taxa (*Firmicutes*, *Bacteroidetes*, *Bifidobacterium*, *Lactobacillus*) (Chae et al., 2024; He et al., 2021; Heida et al., 2016; Lindberg et al., 2020). These patterns are detectable in meconium, highlighting the predictive value of very early colonization (Chae et al., 2024; Heida et al., 2016; Kang et al., 2022). Concomitant metabolic alterations include reduced SCFA (especially butyrate) production (He et al., 2021; Liu X. et al., 2022; Liu Z. et al., 2022) and accumulation of amino acid metabolites and TCA intermediates (e.g., succinate and L-malate) (Chapman et al., 2024; Du et al., 2023). Butyrate deficiency impairs regulatory T-cell induction, disrupting immune homeostasis and exacerbating inflammation (He et al., 2021).

#### 4.1.3 The microbiota-gut-brain axis in the perinatal period

A rapidly expanding area of investigation explores the mechanisms underlying the microbiota-gut-brain axis, particularly during pregnancy and early life. This research posits that microbial disturbances can induce low-grade inflammation and alter immune cell activity, thereby affecting neural circuit development and contributing to behavioral phenotypes such as anxiety, depression, and autism spectrum disorder (ASD). For example, in prenatal depression, reduced Intestinibacter and *Escherichia_Shigella* correlate with elevated IL-1β and IL-17A, implying immune mediated neural effects (Fang et al., 2023; Yang et al., 2022). Prenatal stress (Gur et al., 2017) and high fat diet (Bruce-Keller et al., 2017) induce offspring anxiety and cognitive dysfunction via maternal microbiota alterations. Maternal anxiety (Chi et al., 2024) and alcohol exposure (Wong et al., 2025) reduce microbial diversity and beneficial *Bifidobacterium*, potentially affecting neurodevelopment through IL-6/IL-10 pathways (Galley et al., 2023). Prenatal exposure to valproic acid, LPS, or herbicides induces maternal and offspring dysbiosis alongside ASD-like behaviors, often with sex specific effects (Li H. et al., 2024; Li Y. et al., 2024; Li J. et al., 2024; Li S. et al., 2024; Salia et al., 2025), likely mediated through hormone-immune-microbiome interactions. The vertical transmission of maternal microbiota is a key mechanism, as evidenced by studies showing that infants of mothers with postpartum depression exhibit distinct microbial profiles and poorer neurodevelopmental outcomes (Zhou et al., 2024). Intervention studies, including fecal microbiota transplantation (FMT), have been used to directly demonstrate the microbial contribution to brain development and behavior. Maternal FMT in CS born infants safely restores vaginal delivery like microbiota (Korpela et al., 2020), while FMT from ASD children to mice recapitulates behavioral and neuroinflammatory phenotypes (Avolio et al., 2022). FMT also enhances gut barrier function (e.g., upregulating ZO-1, Occludin) (Zhou et al., 2021) and reduces systemic and neuroinflammation-e.g., by inhibiting LPS-TLR4/NF-κB in arsenic exposure models (Zhao et al., 2023). For instance, in an arsenic exposure model, maternal FMT alleviated offspring neuroinflammation and behavioral deficits by inhibiting the LPS-TLR4/NF-κB pathway (Zhao et al., 2023). The role of microbial metabolites, particularly SCFAs like butyrate, is underscored as a critical signaling pathway facilitating gut-brain communication, immune regulation, and energy metabolism (Chen et al., 2025; Cheng et al., 2019). This cluster represents a paradigm shift toward understanding how the maternal microbial status serves as a modifiable factor in offspring neurodevelopmental programming.

#### 4.1.4 Microecological interventions and translational applications

A highly translational research cluster emphasizes microecological interventions, such as probiotics and prebiotics, for preventing early-life allergic and immune diseases. This area is characterized by a focus on interventional studies, including randomized controlled trials. Evidence suggests that specific probiotic strains can significantly reduce the incidence of NEC in preterm infants (Alfaleh et al., 2010; Chang et al., 2017; Yang et al., 2014) and lower the risk of infant eczema and atopic sensitization when administered to high-risk pregnant women (Barthow et al., 2016). The mechanisms are thought to involve competitive exclusion of pathogens, immune modulation (e.g., restoring Th1/Th2 balance), and enhancement of gut barrier function. Maternal dietary interventions, such as fermentable fiber intake, have also been shown to enrich SCFA-producing bacteria in the offspring and upregulate SCFA receptors (GPR41/43), thereby suppressing allergic airway inflammation (Thorburn et al., 2015; Yuan et al., 2023). This body of work spans a continuous intervention window from pregnancy through early childhood, embodying a preventive medicine approach aimed at shaping the early-life microbiota to reduce long-term disease susceptibility.

### 4.2 Analysis of intellectual foundation and temporal evolution

Analysis of the highly cited and co-cited literature provides insight into the intellectual foundation of the field. Seminal works, such as Koren et al.’s (2012) study demonstrating trimester-specific microbial changes (Koren et al., 2012), and research by Dominguez-Bello et al. (2010) and Backhed et al. (2015) on how delivery mode and feeding practices shape neonatal colonization, form cornerstone references. The frequent co-citation of methodological papers introducing tools like QIIME (Caporaso et al., 2010) and DADA2 (Callahan et al., 2016) underscores the indispensable role of bioinformatic advancements in propelling the field forward. The temporal evolution of research hotspots, as revealed by keyword burst analysis, indicates a clear trajectory. Early research (pre-2010) primarily focused on exploring associations between microbial composition and allergic diseases. As the field matured, themes expanded toward mechanistic investigations of the gut-brain axis, metabolic syndromes, and immune regulation. In recent years, the research frontier has sharpened its focus on specific pregnancy complications (e.g., GDM, preeclampsia), the detailed impact of delivery modes, and the rigorous evaluation of probiotic, prebiotic, and dietary intervention strategies. This evolution signifies a collective shift from initial correlational observations toward a deeper exploration of causal mechanisms and clinical translation.

### 4.3 Global collaboration and key contributors

Research in this field is characterized by robust international collaboration. The China, United States, and Canada lead in terms of publication volume. The United States’ leadership in metrics such as total citations, H-index, and betweenness centrality demonstrates its substantial research accumulation, high-quality output, and central role in coordinating international collaborative networks. While China leads in publication count, its relatively lower average citation rates suggests a future focus on enhancing the international impact of its research output. European nations and Canada also play key hub roles in international collaboration, as evidenced by their advantages in betweenness centrality. Institutional collaboration clustering reveals that the field has formed several efficient collaborative networks centered around regions or research themes, such as North American, European, and Asia-Pacific clusters. This structured collaborative model facilitates resource integration and accelerates knowledge innovation.

A relatively stable and highly productive community of authors has emerged, with several research teams establishing distinctive and influential lines of inquiry. The work of these teams often aligns with and has helped define the major research clusters identified above.

For instance, the team associated with Collado, Maria Carmen; Isolauri, Erika; and Salminen, Seppo has systematically investigated the multifaceted impacts of gut microbiota during pregnancy on maternal and offspring health. Their research has elucidated mechanisms spanning microbial vertical transmission, metabolic-immune regulation, and nutritional interventions. They have confirmed that microbial communities from the maternal gut and breast milk are directly transferred to the newborn, a process influenced by delivery mode and feeding type (Flores Ventura et al., 2025; Saez-Fuertes et al., 2024a; Saez-Fuertes et al., 2024c). Their work has also linked maternal metabolic states, such as overweight/obesity, to gut dysbiosis and systemic inflammation (Zacarias et al., 2018), and explored the potential of probiotic (e.g., *Bifidobacterium breve M-16V*) and synbiotic supplementation (e.g., scGOS/lcFOS) to enhance maternal gut barrier integrity, immune status, and offspring health outcomes (Saez-Fuertes et al., 2024b; Saez-Fuertes et al., 2024c).

The collaborative work of Cryan, John F. and Dinan, Timothy G. has been pivotal in advancing the microbiota-gut-brain axis within the perinatal context. Their research investigates how early-life environmental factors (e.g., stress, antibiotic exposure, diet) affect neurodevelopment via the gut microbiota (Nuzum et al., 2024). Using animal models, they have demonstrated that maternal separation stress induces sex-specific changes in microbiota and leads to long-term visceral hypersensitivity and affective disorders (Wilmes et al., 2024). They have also shown that maternal high-fat diet impacts offspring brain development through microbial-metabolite pathways, such as the kynurenine pathway (Ratsika et al., 2024). Their interventional studies provide experimental support for nutritional and microbial strategies to ameliorate early-life stress-induced behavioral abnormalities (Donoso et al., 2020; McVey Neufeld et al., 2019; O'Mahony et al., 2020; Pusceddu et al., 2015).

Another illustrative example is the team led by Tain, You-Lin; Hsu, Chien-Ning; and Hou, Chih-Yao, which has expounded on how maternal factors (e.g., sweetener intake, chronic kidney disease, high-fat diets) can program offspring hypertension through mechanisms involving oxidative stress, aberrant renin-angiotensin system activity, and gut dysbiosis (Tain et al., 2024a,b; Tain et al., 2024d; Tain and Hsu, 2025). Their research demonstrates the potential of interventions like resveratrol butyrate esters (Tain et al., 2024d), chondroitin sulfate (Tain et al., 2024c), and specific probiotics to reshape microbial structure and prevent hypertensive programming in offspring through pathways involving hydrogen sulfide and nitric oxide signaling (Tain et al., 2023a, 2023b; Tain et al., 2024a,b,c,d).

### 4.4 Strengths, limitations, and future directions

The main methodological contribution of this study is its comparative analysis using both the WoSCC and Scopus databases, which provides valuable insights for bibliometric research practices. The high consistency in annual publication trends between the two databases validates the objective trajectory of the field’s rapid development. However, discrepancies in rankings at the countries/regions, institution, and author levels reflect the different inclusion policies and biases of each database, as previously discussed. Therefore, utilizing both databases allows for a more comprehensive and equitable academic landscape.

Despite these insights, this study has several limitations. Firstly, it primarily focused on journal articles and reviews, excluding conference proceedings, book chapters, or preprints, which might omit some emerging perspectives or data. Secondly, although no formal language restriction was applied, the inherent predominance of English literature in both WoSCC and Scopus means that significant contributions from non-English-speaking regions may not be fully captured, potentially introducing a linguistic and geographical bias. Furthermore, bibliometrics is inherently a quantitative science; it can reveal macro-trends and structural patterns but cannot replace in-depth qualitative assessment of specific research findings or judgments on scientific rigor and validity.

Based on the synthesized research landscape, future directions are likely to focus on several key areas. First, there will be a continued push toward utilizing integrated multi-omics analyses (metatranscriptomics, metabolomics, and proteomics) to move beyond correlation and deeply elucidate the specific molecular pathways through which the gut microbiota influences pregnancy outcomes and offspring health. Second, promoting large-scale, well-designed randomized controlled trials based on existing mechanistic evidence will be crucial to verifying the efficacy and safety of probiotics, prebiotics, or dietary interventions in preventing pregnancy complications and improving long-term offspring health. Third, investigating microbiota characteristics across diverse geographical, ethnic, and socioeconomic populations is essential to ensure that findings are globally applicable and to promote health equity. Finally, exploring the use of microbiota-derived signatures as biomarkers for the early diagnosis and risk prediction of pregnancy complications represents a promising avenue for developing personalized microecological modulation strategies.

The future prospects of maternal and infant microbiome research are profoundly significant. This field holds the potential to fundamentally reshape preventive medicine in the perinatal period. By elucidating the critical role of microbial ecosystems in programming health and disease, it paves the way for novel, non-invasive strategies to improve pregnancy outcomes, optimize infant development, and reduce the long-term burden of non-communicable diseases across the lifespan. As research transitions from observation to intervention, the focus will increasingly be on translating this knowledge into actionable clinical guidelines and public health initiatives that support the microbial health of mothers and their children from pregnancy through the first critical years of life.

## 5 Conclusion

This study demonstrates that database selection significantly influences research assessment outcomes, underscoring the necessity of cross-database analysis for comprehensive evaluation. Through bibliometric analysis of the “gut microbiota during pregnancy” field, we identified its evolution from descriptive studies to mechanistic investigations and clinical applications. Current research focuses on microbial structure, disease mechanisms affecting maternal–infant health, and clinical implications, particularly regarding allergic and metabolic disorders. The mother-infant microbial transmission and gut-brain axis regulation have emerged as crucial areas, highlighting microbiome-host interactions in lifelong health. Emerging frontiers include pregnancy complications (e.g., GDM, preeclampsia), delivery mode impacts, and probiotic/prebiotic interventions. These advancements deepen our understanding of microbial functions during gestation and provide a theoretical foundation for precision nutrition and early-life interventions.

## Data Availability

All bibliometric data analyzed in this study are publicly available from the Web of Science Core Collection and Scopus databases. Processed datasets generated for analysis are included in the article. Further inquiries can be directed to the corresponding author.
